# Exposure to Mono-n-Butyl Phthalate in Women with Endometriosis and Its Association with the Biological Effects on Human Granulosa Cells

**DOI:** 10.3390/ijms21051794

**Published:** 2020-03-05

**Authors:** Ya-Ching Chou, Yu-Chun Chen, Ming-Jer Chen, Ching-Wen Chang, Guan-Lin Lai, Chii-Ruey Tzeng

**Affiliations:** 1 Department of Biological Science and Technology, College of Biological Science and Technology, National Chiao Tung University, Hsinchu 300193, Taiwan; ycchou1@nctu.edu.tw; 2 Center for Intelligent Drug Systems and Smart Bio-devices (IDS2B), National Chiao Tung University, Hsinchu 300193, Taiwan; 3 Department of Obstetrics and Gynecology, School of Medicine, College of Medicine, Taipei Medical University, Taipei 110301, Taiwan; yu150211@gmail.com; 4 Center for Reproductive Medicine & Sciences, Department of Obstetrics and Gynecology, Taipei Medical University Hospital, Taipei 110301, Taiwan; ching967@yahoo.com.tw; 5 Department of Obstetrics and Gynecology and Women’s Health, Taichung Veterans General Hospital, Taichung 407752, Taiwan; mingjerchen@gmail.com; 6 School of Medicine, National Yang-Ming University, Taipei 112304, Taiwan; 7 School of Public Health, College of Public Health, Taipei Medical University, Taipei 110301, Taiwan; m513091008@tmu.edu.tw

**Keywords:** phthalate, mono-*n*-butyl phthalate (MnBP), endometriosis, granulosa cells, mitochondrial membrane potential, anti-Mullerian hormone

## Abstract

To study the association between urinary phthalate metabolite levels, endometriosis, and their effects on human granulosa cells, we recruited patients who underwent laparoscopy to confirm endometriosis (n = 123) and control patients (n = 78). Liquid chromatography–tandem mass spectrometry was used to measure the following five urinary phthalate metabolites: mono-n-butyl phthalate (MnBP), mono(2-ethylhexyl) phthalate, monobenzyl phthalate, mono(2-ethyl-5-oxo-hexyl) phthalate, and mono(2-ethyl-5-hydroxyhexyl) phthalate. Urinary MnBP levels were higher in patients with endometriosis than in controls after multivariable logistic regression including the number of deliveries, body mass index, and use of medicine as covariables. MnBP correlates with other phthalate metabolites. Previous studies found that endometriosis was a detrimental condition for granulosa cells. In our study, we observed whether MnBP affected granulosa cells. MnBP treatment altered the gene expression of BIRC5, BUB1B, CDC20, cyclin B1, IL-1β, TNF-α, inhibin-B, StAR, and P450ssc and attenuated the ratio of the mitochondrial membrane potential in human granulosa cells. Moreover, MnBP decreased the expression of the anti-Mullerian hormone. These findings suggest that MnBP concentration is associated with endometriosis and may affect the health and steroidogenesis of human granulosa cells.

## 1. Introduction

Endometriosis is a common gynecological disorder that is characterized by the presence of endometrial tissue outside the uterus. The prevalence of endometriosis is around 6%–10%, and the disorder may cause 30%–50% of all observed infertility in women [1]. Endometriosis is associated with chronic pelvic pain, dysmenorrhea, menorrhagia, and, in some cases, infertility [2]. Although endometriosis is a relatively common condition, the etiology remains to be elucidated [3]. Emerging evidence suggests that several environmental contaminants may be associated with the pathophysiology of endometriosis [4,5].

Recently, several endocrine-disrupting chemicals (EDCs) have been identified that may contribute to the pathogenesis of endometriosis [6]. These EDCs interfere with hormonal homeostasis and cause changes in estrogen signaling [7,8]. Evidence from animal studies suggest that phthalates possess endocrine-disrupting properties by altering or antagonizing the actions of sex steroid hormones [9,10,11,12,13,14,15,16]. Phthalates are frequently used in the manufacturing of a variety of products in our daily life, including medical devices, food packaging, children’s toys, and polyvinyl chloride plastics [17,18,19]. Consequently, humans have extensive interactions with phthalate-containing items. Exposure to phthalates occurs mainly through contaminated food, packaging materials, pharmaceuticals, or direct contact [20,21,22]. Phthalates are rapidly metabolized and can be detected in blood, breast milk, and urine [21,23,24]. Phthalate metabolites are detected in the United States population [25].

There is increasing evidence linking phthalates to human fertility and reproductive health via their effects on testicular function and spermatogenesis in males [26]. Animal studies have found that exposure to di(2-ethylhexyl) phthalate (DEHP) and its metabolite, mono(2-ethylhexyl) phthalate (MEHP), results in decreased serum progesterone levels, smaller preovulatory follicles or delayed ovulation, anovulation, and increased serum follicle-stimulating hormone (FSH) levels in sexually mature female rats [13,16,27,28].

Several reports have suggested that phthalate may be a risk factor for endometriosis [5,29,30,31,32,33,34,35]. However, most of these studies have limitations, including the detection of phthalate metabolites in plasma, inclusion of many confounders, selection bias, and small study populations. In this study, we aimed to investigate the association between phthalate levels and endometriosis. According to previous studies [5,29,30,31,32,33,34,35], we chose and used high-performance liquid chromatography–tandem mass spectrometry to detect five phthalate metabolites: Mono-n-butyl phthalate (MnBP), MEHP, monobenzyl phthalate (MBzP), mono(2-ethyl-5-oxo-hexyl) phthalate (MEOHP), and mono(2-ethyl-5-hydroxyhexyl) phthalate (MEHHP).

Recently, DEHP-treated endometrial cells caused cell invasion, increased matrix metalloproteinase-2 (MMP2) and MMP9 activities, and increased p21-acticated kinase 4 expression [36]. In non-obese diabetic/severe combined immunodeficiency (NOD/SCID) mice fed with DEHP, the endometrial implant volume increased, suggesting that exposure to phthalate may cause endometriosis [36]. Endometriosis may decrease steroidogenesis, reduce aromatase activity, alter cell cycle progression, change mitochondrial gene expression, and increase tumor necrosis factor α expression in granulosa cells [37,38,39,40,41]. These reports suggested that endometriosis may be detrimental to granulosa cells [42]. Moreover, a previous study detected MnBP in human follicular fluid [43]. Mural granulosa cells and theca cells play a crucial role in oocyte development, ovarian follicular growth, and steroidogenesis. Several reports have shown that phthalate metabolites may affect the granulosa cells. In rat granulosa cells, MEHP suppresses aromatase transcript levels, estradiol production, steroid hormone synthesis, and key enzymes in progesterone production, and causes the attenuation of cell proliferation and induction of apoptosis [16,44,45]. These results suggest that MEHP has important effects on rodent granulosa cells, but there is no information in humans. Furthermore, endometriosis is a harmful condition for human granulosa cells [42]. Therefore, after investigating the associations between phthalate metabolites and endometriosis, we examined the effects of phthalate metabolites on human granulosa cells.

## 2. Results

### 2.1. Patient Demographics

The patient demographics are shown in Table 1. There were significant differences in the body mass index (BMI) and number of deliveries between the endometriosis and control groups. The mean BMI was 20.6 (standard deviation (SD): 3.4) kg/m^2^ in the endometriosis group and 22.1 (SD: 3.6) kg/m^2^ in the controls. The numbers of deliveries were 0.3 (SD: 0.6) in the endometriosis group and 0.5 (SD: 0.9) in the control group. The rate of dysmenorrhea and use of medicine increased in women with endometriosis. Women with endometriosis were taking more medicine than the control group, which may have been for easing painful menstrual cramps. The two groups did not differ significantly in age at menarche (*p* = 0.559), regulation of the menstrual cycle (*p* = 0.544), cigarette smoking status (*p* = 0.222), or regulation of the menstrual cycle (*p* = 0.302) (Table 1).

### 2.2. Comparison of Urinary Phthalate Metabolites between the Endometriosis and Control Groups

We next used high-performance liquid chromatography–tandem mass spectrometry to measure the concentrations of phthalate metabolites After controlling for potential confounders, the geometric mean MnBP concentration was significantly higher in endometriosis patients (148.4 (SD: 136.8) μg/g creatinine) than in the controls (109.9 (SD: 175.5) μg/g creatinine) (Table 2). A higher MnBP concentration correlated with the presence of endometriosis (odds ratio (OR): 1.89; 95% confidence interval (CI): 1.05–3.39). These results suggest that MnBP concentration was found to be associated with endometriosis.

### 2.3. Correlations between Phthalate Metabolites in Human Urine

**Figure 1:**
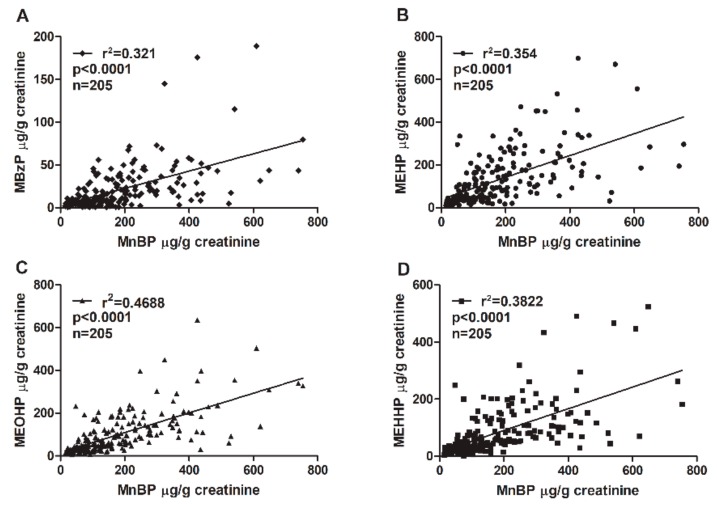
Correlations between urinary MnBP concentration and the concentrations of (**A**) MEHP, (**B**) MBzP, (**C**) MEOHP, and (**D**) MEHHP. The total sample size was 205. The coefficient of determination (*r*^2^) was (A) 0.321, (B) 0.354, (C) 0.4688, and (D) 0.3822. All correlations had *p* values < 0.0001. Mono-n-butyl phthalate (MnBP), mono(2-ethylhexyl) phthalate (MEHP), monobenzyl phthalate (MBzP), mono(2-ethyl-5-oxo-hexyl) phthalate (MEOHP), and mono(2-ethyl-5-hydroxyhexyl) phthalate (MEHHP).

The phthalate diesters are metabolized into primary hydrolytic and secondary oxidative monoester metabolites (Table 3). MnBP is the major metabolite of dibutyl phthalate (DBP). Benzyl butyl phthalate (BzBP) is metabolized into MBzP and a small portion of MnBP [46]. An association between MnBP and MBzP concentrations was confirmed. The coefficient of determination (*r*^2^) between MnBP and MBzP was 0.321 (Figure 1). Other phthalate metabolites were also analyzed. The concentration of MnBP metabolites correlated significantly with those of other phthalate metabolites measured in urine (Figure 1). The coefficient of determination (*r*^2^) between MnBP and MEHP, MEOHP, and MEHHP was 0.354, 0.4688, and 0.3822, respectively. All *p* values were <0.0001 (Figure 1). These results suggest that women with endometriosis who have been exposed to a high level of DBP or BzBP may have also been exposed to other phthalates.

### 2.4. Effects of MnBP on the Expression of BIRC3, BUB1B, CDC20, and Cyclin B1 

**Figure 2:**
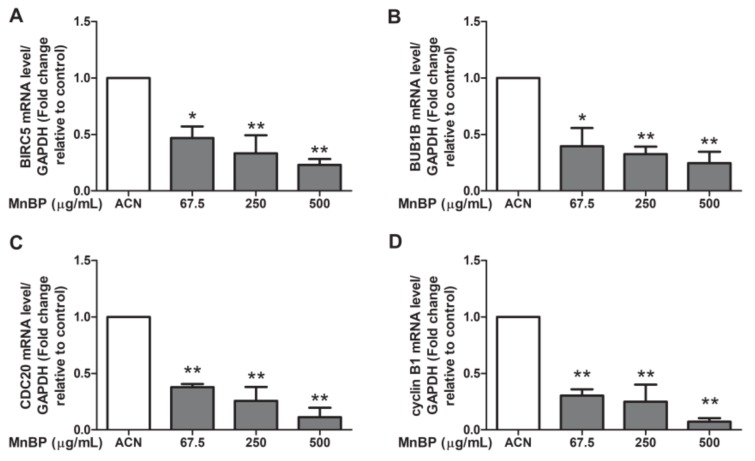
Effects of MnBP treatment on the expression of (**A**) *BIRC5*, (**B**) *BUB1B*, (**C**) *CDC20*, and (**D**) cyclin B1 in human granulosa cells. The expression levels of mRNA for the baculoviral inhibitor of apoptosis repeat-containing 5 (*BIRC5*), mitotic checkpoint serine/threonine kinase beta (*BUB1B*), cell division cycle 20 (*CDC20*), and cyclin B1 were measured using the reverse transcription quantitative polymerase chain reaction. Human granulosa cells were seeded overnight and treated with solvent control (ACN) and 67.5, 250, or 500 µg/mL of MnBP for 24 h. The expression levels of *BIRC5*, *BUB1B*, *CDC20*, and cyclin B1 were normalized to that of glyceraldehyde-3-phosphate dehydrogenase (GAPDH). Data are shown as mean ± SD compared to those of the solvent control. * *p* < 0.05. ** *p* < 0.01.

A recent study reported that DBP can affect the cell cycle, mitosis, Rho GTPase, Aurora B signaling pathway, polo-like kinase 1, and E2F-mediated DNA replication in human mural granulosa cells from the functional annotation of differentially expressed genes [47]. Moreover, the expression levels of *BIRC3*, *BUB1B*, *CDC20*, and cyclin B1 are decreased in DBP treated cells [47]. We found that MnBP is associated with endometriosis and is the primary metabolite of DBP. Therefore, we analyzed DBP-regulated genes in human granulosa cells after exposure to MnBP. However, there were too few granulosa cells to collect from the endometriosis and control groups and to seed on the same day, so the collected granulosa cells were pooled on the oocyte retrieval day. The gene expression levels of *BIRC3*, *BUB1B*, *CDC20*, and cyclin B1 were lower than those of the untreated cells (Figure 2). These results suggest that MnBP decreased the *BIRC3*, *BUB1B*, *CDC20*, and cyclin B1 gene expression levels. These genes are responsible for regulation of the G2/M phase of mitosis and spindle assembly checkpoint [48,49,50,51,52].

### 2.5. Effect of MnBP on the Expression of IL-1β and TNF-α

**Figure 3:**
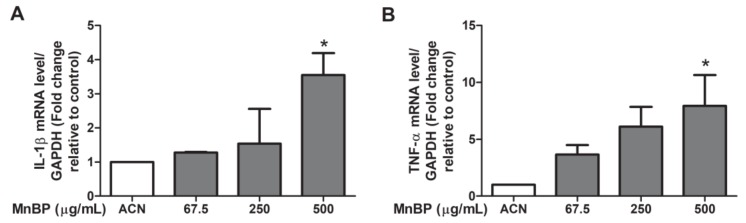
Effects of MnBP treatment on the expression of (**A**) IL-1β and (**B**) TNF-α. The expression levels of mRNA for the interleukin-1β (IL-1β) and tumor necrosis factor-α (TNF-α) were measured using the reverse transcription quantitative polymerase chain reaction. Human granulosa cells were seeded overnight and treated with solvent control (ACN) and 67.5, 250, or 500 µg/mL of MnBP for 24 h. The expression levels of IL-1β and TNF-α were normalized to that of GAPDH. Data are shown as mean ± SD compared to those of the solvent control. * *p* < 0.05.

Previous studies have showed that IL-1β, IL-6, and TNF-α are increased in women with endometriosis [53]. We found that MnBP increased the IL-1β and TNF-α gene expression levels (Figure 3). The expression of IL-6 was not affected by MnBP treatment (data not shown).

### 2.6. Effect of MnBP on Human Granulosa Cell Mitochondrial Membrane Potential

**Figure 4:**
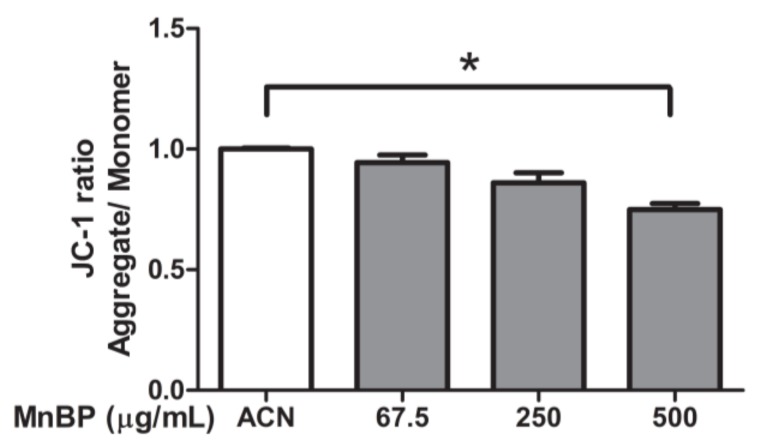
Effects of MnBP on the ratio of mitochondrial membrane potential in human granulosa cells. Human granulosa cells were seeded overnight in 96-well plates and treated with solvent control (ACN) and 67.5, 250, or 500 µg/mL of MnBP for 72 h. The treated cells were stained with JC-1 solution (a cationic dye) and incubated at 37 °C in 5% CO_2_ for 30 min. The ratio of the fluorescence intensity of J-aggregates (excitation and emission at 550 and 600 nm, respectively) to that of J-monomer (excitation and emission at 485 and 535 nm, respectively) was used as the indicator of cell health. * *p* < 0.05.

A previous study detected MnBP in human follicular fluid [43]. The effects of MnBP on mitochondrial function in human granulosa cells were assessed by measuring the ratio of the fluorescence intensity of J-aggregates to that of monomers and was used as an indicator of cell health. Exposure to MnBP significantly decreased the ratio of the mitochondrial membrane potential (Figure 4).

### 2.7. Effect of MnBP on AMH, Inhibin B, StAR, and P450ssc Expression 

**Figure 5:**
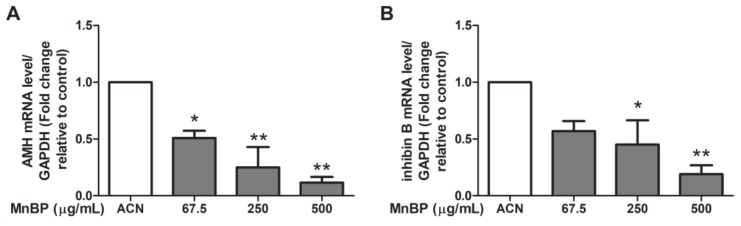
Effects of MnBP on the expression of (**A**) AMH and (**B**) inhibin B in human granulosa cells. The expression levels of mRNA for the AMH and inhibin B was measured using the reverse transcription quantitative polymerase chain reaction. Human granulosa cells were seeded overnight and treated with solvent control (ACN) and 67.5, 250, or 500 µg/mL of MnBP for 24 h. The expression levels of *AMH* and *inhibin B* were normalized to that of GAPDH. Data are shown as mean ± SD compared to those of the solvent control. * *p* < 0.05. ** *p* < 0.01.

Recently, a negative association between urinary phthalate concentrations and serum inhibin B levels was discovered [54]. Endometriosis also decreases the secretion of inhibin B during ovarian stimulation [55]. As endometriosis is detrimental to granulosa cells [42], we analyzed the expression of AMH in human granulosa cells. Figure 5 shows that MnBP treatment decreased the level of AMH and inhibin B in human granulosa cells. 

**Figure 6:**
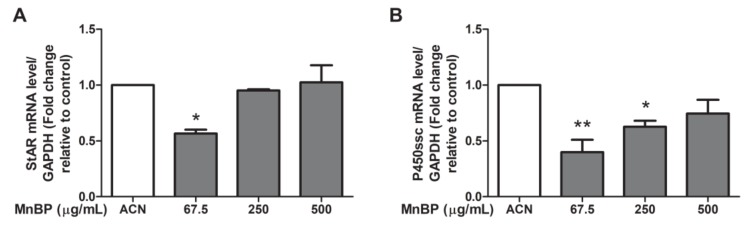
Effects of MnBP on the expression of (**A**) StAR and (**B**) P450ssc in human granulosa cells. The expression levels of mRNA for the steroidogenic acute regulatory protein (StAR) and cytochrome cholesterol side-chain cleavage enzyme (P450scc) were measured using the reverse transcription quantitative polymerase chain reaction. Human granulosa cells were seeded overnight and treated with solvent control (ACN) and 67.5, 250, or 500 µg/mL of MnBP for 24 h. The expression levels of *StAR* and *P450ssc* were normalized to that of GAPDH. Data are shown as mean ± SD compared to those of the solvent control. * *p* < 0.05. ** *p* < 0.01.

Moreover, we test MnBP exposure on altering the expression of the gene coding protein and enzyme that are associated with steroidogenesis in human granulosa cells, including steroidogenic acute regulatory protein (StAR) and cytochrome cholesterol side-chain cleavage enzyme (P450scc). The expressions of StAR and P450scc were lower after exposure to MnBP (Figure 6). These results are similar to those of the exposure of MEHP in rat ovarian granulosa cells and the exposure of DBP in human granulosa cells at low dose [45,48]. 

## 3. Discussion 

In this study, high-performance liquid chromatography–tandem mass spectrometry was used to identify which phthalate metabolites were related to endometriosis. Multiple logistic regression analysis showed that a higher MnBP concentration was significantly associated with the occurrence of endometriosis (Table 2). MnBP levels correlated strongly with the levels of other phthalate metabolites (Figure 1).

In addition, MnBP affected the gene expression levels of *BIRC3*, *BUB1B*, *CDC20*, cyclin B1, IL-1β, and TNF-α and attenuated the ratio of the mitochondrial membrane potential in human granulosa cells (Figure 2, Figure 3 and Figure 4). MnBP also decreased the expression of AMH and inhibin B (Figure 5). These results suggest that MnBP is associated with endometriosis and may affect the ratio of the mitochondrial membrane potential, AMH, inhibin B, StAR, and P450ssc expression of human granulosa cells. 

Endometriosis can cause infertility in women. In our study, women with endometriosis reported fewer deliveries (Table 1). The prevalence of endometriosis is lower in pregnant than nonpregnant women, which suggests that pregnancy may protect against endometriosis [56]. Recurrence of endometriosis within 2–6 years is less common in pregnant than nonpregnant women [57,58,59]. Our finding of a lower BMI in women with endometriosis than controls (Table 1) is consistent with the findings of other studies [60,61]. Women with endometriosis were taking more medicine than the control group (Table 1), which may have been for the pain relief of menstrual cramps. We discovered that MnBP levels correlated with the levels of other phthalate metabolites, including MEHP, MBzP, MEHHP, and MEOHP. In Table 2, we show that co-exposure to DEHP and DBP as their metabolites MEHP and MnBP was higher in women with endometriosis than controls, which implies that women with endometriosis are found to not only be exposed to DBP but also DEHP simultaneously. 

Accumulating evidence suggests that extracellular matrix metalloproteinase, adhesion molecules, immune cells, and pro-inflammatory cytokines activate or alter the peritoneal microenvironment, which creates the conditions for adhesion, differentiation, proliferation, and survival of ectopic endometrial cells. It is marked by an inflammatory state associated with inflammatory mediators. Recently, it has been shown that epigenetic mechanisms control biological aberrations in endometriosis, and the heterogeneity of endometriosis suggests that it is a complex disease and a single model is not sufficient for explaining its pathogenesis [62,63,64,65]. Environmental toxins may be important issues of endometriosis. 2,3,7,8-Tetrachlorodibenzo-p-dioxin (TCDD) is lipophilic and accumulates in the fat tissue and in the food chain. Current epidemiological studies find that the association between TCDD and endometriosis remains controversial. TCDD also causes epigenetic modifications [66,67]. Further studies focusing on the environmental toxins and epigenetic mechanisms in endometriosis are required to determine the pathogenesis of endometriosis. 

Several studies have demonstrated an association between endometriosis and the levels of phthalate metabolites [5,29,31,32,33,34,35,68]. Our study has some advantages over these previous studies. For example, we collected reliable data from medical records and included patients with laparoscopy-confirmed endometriosis and controls, which reduced the risk of potential selection bias due to the exclusion of patients with non-laparoscopy-confirmed results. We also adjusted for important potential confounders that may influence phthalate exposure in women with endometriosis. The use of medicine was higher in women with endometriosis than in the control group (Table 1). That may have been due to the pain relief for menstrual cramps and may have been an additional source of phthalate exposure. Thus, we adjusted for the use of medicine in logistic regression analysis. These findings suggest that urinary MnBP levels were found to coincide with endometriosis. 

Previous studies have shown that MnBP, the main metabolite of DBP, and other phthalate metabolites, including monomethyl phthalate, monoethyl phthalate, and monopropyl phthalate, have no effects on estradiol synthesis [16]. Using high-performance liquid chromatography to measure DEHP and MEHP concentrations, Cobellis et al. found that plasma DEHP concentrations were significantly higher in women with endometriosis than in healthy controls [31]. In India, higher blood levels of DBP, BzBP, di-*n*-octyl phthalate, and DEHP were detected in women with endometriosis compared to women free from endometriosis [30,68]. In South Korea, using liquid chromatography–tandem mass spectrometry, Kim et al. found significantly higher plasma concentrations of MEHP and DEHP in women with endometriosis than in controls [34]. 

Most studies measured the plasma or serum levels of phthalates and/or phthalate metabolites as an indicator of exposure. However, these methods to estimate exposure have several limitations. The detection of phthalates in body fluids can be confounded by contamination from phthalate-containing laboratory supplies. Moreover, serum enzymes can hydrolyze diesters to monoesters, which can lead to false-positive results [69]. Phthalates can be ingested from contaminated food or breathed in via air that contains phthalate vapor or dust contaminated with phthalate particles. After entering the body, diester phthalates are rapidly hydrolyzed to monoester phthalates, while some of the more nonpolar phthalates are further oxidized. Both the hydrolyzed and oxidized metabolites are also conjugated by glucuronidation before excretion in urine [70]. Consequently, phthalate exposure among the general population can be detected using the stable phthalate metabolites in urine as a biomarker [71]. Itoh et al. found no association between the urinary concentration of monoethyl phthalate (MEP), MnBP, MBzP, MEHP, MEHHP, and MEOHP in 57 Japanese women with endometriosis and 80 controls [29]. The inconsistent results may reflect differences in sample size, adjustment for confounders, and variation in exposure between different areas. 

In a study from the United States, the urinary MnBP level was associated with self-reported endometriosis [32]. Another study reported that urinary MEHP concentration was associated with endometriosis when compared to women who were diagnosed with a normal pelvis after surgery [5]. Upson et al. found that MBzP and MEP may increase the risk of endometriosis [35]. Furthermore, higher log-transformed urinary concentrations of MEHHP and MEOHP are associated with endometriosis in Korean women [36]. 

A recent study showed that treatment of endometrial cells with DEHP in vitro increased matrix metalloproteinase 2 (MMP-2) and MMP-9 activities, induced cell invasion, and increased endometrial implantation in nonobese diabetic/severe combined immunodeficiency mice [36]. Genetic analysis after phthalate exposure revealed that the glutathione S-transferase M1 (a major detoxification enzyme) null genotype and phthalate exposure were associated with increased risk for adenomyosis and leiomyoma [33]. 

The effects of DBP on rat granulosa cells have shown that DBP does not induce cell death or affect cell proliferation [72]. DBP also reduces aromatase expression and FSH-induced estradiol and progesterone levels [72]. In human mural granulosa cells, DBP impairs the expression of luteinizing hormone-dependent genes [48]. A recent study of global gene expression of human granulosa cells treated with DBP reported that DBP exposure affects the expression of several genes, including *CDC20*, *BIRC5*, *BUB1B*, and cyclin B1 [48]. 

We also found that exposure to MnBP affected the gene expression of *CDC20*, *BIRC5*, *BUB1B*, *cyclin B1*, *IL-1β*, and *TNF-α* (Figure 2 and Figure 3). Previous studies have shown that *BIRC5* mRNA expression was elevated in granulosa cells, particularly during the G2/M phase of mitosis. *BUB1B*, *CDC20*, and cyclin B1 play key roles in the spindle assembly checkpoint [49,50,51,52]. In our study, MnBP significantly affected the expression levels of *CDC20*, *BIRC5*, *BUB1B*, cyclin B1, IL-1β, and TNF-α, and decreased the ratio of the mitochondrial membrane potential (Figure 2, Figure 3 and Figure 4). DBP does not affect the proliferation of human mural granulosa cells [48]. However, in our study, MnBP affected cell health in human granulosa cells (Figure 4). MnBP is also the minor metabolite of BzBP. Chen et al. found that BzBP increased the expression levels of the aryl hydrocarbon receptor, aryl hydrocarbon receptor nuclear translocator, and cytochrome-P450 (CYP)1B1, thereby inducing cell necrosis in immortalized HO23 human granulosa cells [73].

In our study, the pooled human granulosa cells were collected during oocyte retrieval, not from women with endometriosis and controls. The current study had difficulty enrolling endometriosis patients and controls on the same day to compare the biological effects of granulosa cells. Further studies using granulosa cells from endometriosis patients and controls are required to determine the direct association of MnBP and endometriosis in human granulosa cells. 

In this study, we found that MnBP affected cell health, AMH, and inhibin B production, while these cells were not collected from women with endometriosis and the control groups. Thus, the current study had a limited connection between endometriosis, MnBP, and the effects of MnBP and endometriosis on granulosa cells. Therefore, further studies with granulosa cells, which were collected separately from women with endometriosis and controls, will strengthen the molecular mechanism of the effects of MnBP on granulosa cells in women with endometriosis compared to controls. The current study had a limited number of urinary samples; therefore, further studies with larger sample sizes are required. Moreover, the control group in this study was women with other gynecological conditions that required surgery, which may cause other potential issues.

We measured the concentrations of five urinary phthalate metabolites and investigated their association with endometriosis. MnBP levels correlated with the presence of endometriosis (OR = 1.89). All women enrolled in this study were of the same ethnic background and lived in a similar geographic location, which reduced the potential variance derived from the genetic and environmental backgrounds. We found a geometric mean MnBP concentration of 148.4 (SD: 136.8) μg/g creatinine in the endometriosis patients and 109.9 (SD: 175.5) µg/g creatinine in the control patients. The high MnBP level may reflect a higher exposure level in Taiwan [74]. Our findings strongly suggest that urinary MnBP was associated with endometriosis, and MnBP can affect gene expression and attenuate the ratio of the mitochondrial membrane potential, AMH, and inhibin B production in human granulosa cells. 

## 4. Materials and Methods 

### 4.1. Subject Recruitment

We recruited 205 patients who underwent laparoscopic confirmation of endometriosis (*n* = 123) or who had other gynecological diseases (controls, *n* = 82) in the Center for Reproductive Medicine at Taipei Medical University Hospital from 2013 to 2016. All women with endometriosis were diagnosed as stage III or IV endometriosis. The indication in the control groups for laparoscopy included myomas, fibrous adhesions, teratomas, hydrosalpinx; and ovarian, dermoid, paratubal, luteum, and simple cysts. All diagnoses were confirmed by pathological analysis and divided into endometriosis and control groups. Informed consent was obtained from each patient, and the study was approved by the Taipei Medical University Joint Institutional Review Board (TMU-JIRB 201305035, TMU-JIRB 201405030 and TMU-JIRB 201410033). Diagnosis of endometriosis was confirmed by histological analysis, and patients without endometriosis were included as controls. A trained researcher interviewed subjects using a structured questionnaire, and specialized medical staff collected a urine specimen from each patient before the laparoscopy. The structured questionnaire sought information such as education, number of deliveries, body mass index (BMI), age at menarche, duration of menstrual cycle, dysmenorrhea, regulation of menstrual cycle, and use of medicine. All participants were Han Chinese women. The granulosa cells were routinely aspirated and collected during oocyte retrieval from women who underwent in vitro fertilization.

### 4.2. Measurement of Urinary Phthalate Metabolite Concentrations

Urine samples (8–10 mL) were collected, immediately centrifuged, and stored at –80 ℃ until further laboratory analysis. We measured the concentrations of five phthalate metabolites: MnBP, MEHP, MBzP, MEOHP, and MEHHP. In this study, plastic equipment was not used in any experimental process to prevent contamination. All glass apparatuses were washed and rinsed with deionized water and methanol before drying. To measure phthalate concentrations, the frozen samples were thawed and sonicated for 10 min. From each urine sample, 1 mL was mixed with 250 μL of ammonium acetate (1 M, pH = 6.5) and spiked with a mixture of ring isotope-labelled phthalate monoester standards (37.5 ng/mL; LGC Ltd., Teddington, UK) and 5 μL of β-glucuronidase (from E.coli K12, Roche, IN, USA). After incubation at 37 °C for 90 min in a shaker water bath, the samples were diluted with 5 mL of formic acid (0.1 M; Honeywell, NM, USA). The mixture was then purified using solid-phase extraction (SPE) and eluted with 1 mL of H_2_O and 2 mL of 10% methanol. Finally, 1 mL of acetonitrile (ACN; JT baker, NJ, USA) was added to the SPE, and the mixture was collected and stored at –20 ℃ until further analysis. The concentrations of monoester phthalates were measured using high-performance liquid chromatography–tandem mass spectrometry. The chromatographic resolution was accomplished using a 3 μm, 150 mm × 2 mm column (Luna 3 μm Phenyl-Hexyl 100 Å, LC Column 150 × 2 mm; Phenomenex, Torrance, CA, USA) and operated at a 0.5 mL/min flow rate with 0.1% acetic acid in H_2_O as mobile phase A and 0.1% acetic acid in ACN as mobile phase B. Mobile phases A and B were delivered at a flow rate of 0.5 mL/min according to the following gradient: 0 min: 50 (A)/50 (B)%; 5 min: 20 (A)/80 (B)%; 8 min 0 (A)/100 (B)%; and 12 min: 50 (A)/50 (B)%. The phthalate metabolites were normalized with urinary creatinine and the geometric mean was calculated in the endometriosis and control groups. 

### 4.3. Cell Culture

In women who underwent in vitro fertilization (ages 28 to 39 years old), granulosa cells were routinely aspirated and collected during oocyte retrieval. As there were too few granulosa cells to collect from endometriosis and control groups and to seed on the same day, the collected granulosa cells were pooled on the oocyte retrieval day and seeded in 12- or 24-well plates overnight. The cells were cultured in McCoy’s 5A medium (Sigma-Aldrich, London, UK) with l-glutamine, sodium bicarbonate, 10% fetal bovine serum (Gibco, Thermo Fisher Scientific, Waltham, MA, USA), and 1% penicillin/streptomycin/amphotericin B (Biological Industries, Cromwell, CT, USA) at 37 °C with 5% CO_2_. As the MnBP was dissolved in acetonitrile (ACN), we used ACN as the solvent control in the non-treatment control group. For the MnBP treatment, 1 mL culture medium was replaced and supplemented with 1 μL of solvent control (ACN), or 1 μL of 67.5, 250, or 500 µg/mL of MnBP for 24 h in the reverse transcription quantitative polymerase chain reaction (RT-Q-PCR) assay, or for 72 h in the analysis of mitochondrial membrane potential. For all treatments with MnBP, MnBP was dissolved in the same volume (1 µL) of ACN to exclude the effects of volume differences on granulosa cells. The cells were treated with different concentrations of MnBP but with the same volume of solvent (ACN).

### 4.4. Reverse Transcription Quantitative Polymerase Chain Reaction (RT-Q-PCR)

Human granulosa cells were treated with the solvent control (ACN) and 67.5, 250, or 500 µg/mL of MnBP for 24 h. Total RNA was purified from treated human granulosa cells via an RNeasy Plus Mini Kit (Qiagen, Germantown, MD, USA) according to the manufacturer’s instructions. RNA concentration was measured using a NanoDrop 2000/c instrument (Thermo Fisher Scientific, Waltham, MA, USA), and the A260/A280 ratio was more than 1.9 for each sample. Reverse transcription was performed using an iScript cDNA synthesis kit (Bio-Rad, Hercules, CA, USA) according to the manufacturer’s instructions. The quantitative polymerase chain reaction (q-PCR) was conducted using 2× sensiFAST SYBR Hi-ROX Mix (Bioline, Taunton, MA, USA) with the specific primers in Table 4. 

### 4.5. Assay to Measure Mitochondrial Membrane Potential

Human granulosa cells were treated with the solvent control (ACN) and 67.5, 250, or 500 µg/mL of MnBP for 72 h. The 200 μM JC-1 (a cationic dye) staining solution (Molecular Probes, Thermo Fisher Scientific, Waltham, MA, USA) was added to the culture medium, and the cells were incubated at 37 °C in 5% CO_2_ for 30 min and then harvested. The ratio of the fluorescence intensity of J-aggregates (excitation and emission at 535 and 595 nm, respectively) to that of J-monomer (excitation and emission at 485 and 535 nm, respectively) was used as an indicator of cell health [75].

### 4.6. Statistical Analyses

All analyses were performed using IBM SPSS Statistics, version 20 for Windows (New York, NY, USA). A *p* value <0.05 was considered significant. We used Student’s *t* test for continuous variables to analyze the demographic variables and urinary levels of phthalate metabolites and the χ^2^ test for discontinuous variables. All phthalate metabolite measurements were log-transformed to improve the linearity of the model relationship. Creatinine-corrected concentrations of urinary phthalate metabolites (μg/g creatinine) were used to normalize for the urine dilution. In logistic regression modelling, we adjusted for the main covariates in this study: BMI, number of deliveries, and use of medicine. Correlations between MnBP concentration and other phthalate metabolites were calculated by linear regression using GraphPad Prism (San Diego, CA, USA). The coefficient of determination (*r*^2^) and *p* values were calculated. For the MnBP treatment experiment, the mitochondrial membrane potential and relative gene expression levels of *BIRC5*, *BUB1B*, *CDC20*, cyclin B1, IL-1β, TNF-*α*, AMH, inhibin B, StAR, and P450ssc were analyzed using one-way analysis of variance with GraphPad Prism.

## Figures and Tables

**Table 1:** Clinical characteristics of women with endometriosis and control.

Characteristics	Endometriosis Group	Control Group	*p* Value
Total, n	123	82	
Education			
Junior high school, n (%)	3 (2.4%)	2 (2.4%)	
Senior high school, n (%)	13 (10.6%)	13 (15.9%)	
College/University, n (%)	73 (59.3%)	40 (48.8%)	
Graduate School, n (%)	21 (17.1%)	19 (23.2%)	
No. of deliveries	0.3 (0.6)	0.5 (0.9)	0.045 ^a^
BMI ^a^, kg/m^2^	20.6 (3.4)	22.1 (3.6)	0.002 ^a^
Age of menarche	12.8(1.4)	12.6 (1.2)	0.559 ^a^
Duration of menstrual cycle	28.5 (2.9)	28.8 (4.9)	0.544 ^a^
Dysmenorrhea, n (%)	92 (74.8%)	43 (52.4%)	0.001 ^b^
Regulation of menstrual cycle, n (%)	99 (80.5%)	61 (74.4%)	0.302 ^b^
Cigarette smoking status, n (%)	10 (8.1%)	11 (13.4%)	0.222 ^b^
Use of medicine, n (%)	61 (49.6%)	23 (28.0%)	0.002 ^b^
Oral contraceptive, n (%)	7 (11.5%)	2 (8.7%)	
Progesterone, n (%)	8 (13.1%)	4 (17.4%)	
Leuprolide acetate, n (%)	34 (55.7%)	9 (39.1%)	
Others, n (%)	9 (14.8%)	1 (4.3%)	
Combination of medicine, n (%)	3 (4.9%)	7 (30.4%)	

Abbreviations: BMI, body mass index; SD, standard deviation, Mean (SD) for continuous variables. n (%) for discontinuous variables. ^a^: Student’s *t* test, ^b^: χ^2^ test.

**Table 2:** Urinary levels of phthalate metabolites of endometriosis patients and control.

Creatinine-Adjusted Urine Levels (µg/g Creatinine)	Endometriosis Group (n = 123) ^a^	Control Group (n = 82) ^a^	*p* Value ^b^	*p* Value Adjusted ^c^	OR (95% CI) ^c^
MnBP ^a^	148.4 (136.8)	109.9 (175.5)	0.022 *	0.034 *	1.89 (1.05–3.39)
MEHP ^a^	99.5 (112.8)	73.7 (157.6)	0.069	0.406	1.33 (0.68–2.59)
MBzP ^a^	12.2 (18.1)	10.0 (37.4)	0.244	0.337	1.27 (0.78–2.06)
MEOHP ^a^	66.7 (83.7)	60.3 (127.8)	0.392	0.562	0.73 (0.25–2.15)
MEHHP ^a^	54.6 (69.3)	54.6 (122.4)	0.976	0.182	0.57 (0.25–1.31)

Abbreviations: CI, confidence interval; OR, odds ratio; SD, standard deviation, ^a^ Values are geometric mean (SD), ^b^ Student’s *t* test, ^c^ Adjusted for BMI, number of deliveries, and use of medicine using logistic regression analysis. * *p* < 0.05.

**Table 3:** Phthalate diesters and corresponding urinary phthalate metabolites.

Phthalate Diester	Primary Hydrolytic Monoester Metabolite	Secondary Oxidative Monoester Metabolite
Dibutyl phthalate (DBP)	Mono-n-butyl phthalate (MnBP)	
Di(2-ethylhexyl) phthalate (DEHP)	Mono-(2-ethylhexyl) phthalate (MEHP)	Mono-(2-ethyl-5-hydroxyhexyl) phthalate (MEHHP) Mono-(2-ethyl-5-oxo-hexyl) phthalate (MEOHP) Mono-(2-ethyl-5-carboxypentyl) phthalate (MECCP)
Benzylbutyl phthalate (BzBP)	Mono-benzyl phthalate (MBzP) Mono-n-butyl phthalate (MnBP)	

**Table 4:** Primer lists of BIRC5, BUB1B, CDC20, cyclin B1, IL-1β, TNF-*α*, AMH, inhibin B, StAR, P450ssc, and GAPDH.

Gene Name	Forward Primers	Reverse Primers
baculoviral inhibitor of apoptosis repeat-containing 5 (BIRC5)	5′-cttggcccagtgtttcttct-3′	5′-cttattgttggtttcctttgcat-3′
budding uninhibited by benzimidazoles 1 homolog beta, mitotic checkpoint serine/threonine kinase beta (BUB1B)	5′-tgcatttgaagcccagttt-3′	5′-caaagaagagatgatcttattgactcc-3′
cell division cycle 20 (CDC20)	5′-ctgtctgagtgccgtggat-3′	5′-tccttgtaatggggagacca-3′
cyclin B1	5′- ccagtgccagtgtctgagc-3′	5′-tggagaggcagtatcaacca-3′
interleukin-1β (IL-1β)	5′-atgatggcttattacagtggcaa-3′	5′-gtcggagattcgtagctgga-3′
tumor necrosis factor-*α* (TNF-*α*)	5′-gaggccaagccctggtatg-3′	5′-cgggccgattgatctcagc-3′
anti-Mullerian hormone (AMH)	5′-cgcctggtggtcctacac-3′	5′- gaacctcagcgagggtgtt-3′
inhibin B	5′-ctctgcctggctcgatgt-3′	5′-aggccttgaagcacgaag-3′
steroidogenic acute regulatory protein (StAR)	5′-gggagtggaaccccaatgtc-3′	5′-ccagctcgtgagtaatgaatgt-3′
cytochrome cholesterol side-chain cleavage enzyme (P450scc)	5′-ctgcatgggacgtgattttc-3′	5′-cagggtcatggacgtcgtgt-3′
glyceraldehyde-3-phosphate dehydrogenase (GAPDH)	5′-gagtccactggcgtcttcac-3′	5′-gttcacacccatgacgaaca-3′

## Abbreviations

EDC	endocrine-disrupting chemicals
DEHP	Di(2-ethylhexyl) phthalate
MnBP	Mono-n-butyl phthalate
DBP	Dibutyl phthalate
MEHP	Mono-(2-ethylhexyl) phthalate
MEHHP	Mono-(2-ethyl-5-hydroxyhexyl) phthalate
MEOHP	Mono-(2-ethyl-5-oxo-hexyl) phthalate
MECCP	Mono-(2-ethyl-5-carboxypentyl) phthalate
BzBP	Benzylbutyl phthalate
MBzP	Mono-benzyl phthalate
FSH	follicle-stimulating hormone
BMI	body mass index
IL-1β	interleukin-1β
TNF-α	tumor necrosis factor-α
BIRC5	baculoviral inhibitor of apoptosis repeat-containing 5
BUB1B	budding uninhibited by benzimidazoles 1 homolog beta, mitotic checkpoint serine/threonine kinase beta
CDC20	cell division cycle 20
AMH	anti-Mullerian hormone
StAR	steroidogenic acute regulatory protein
P450scc	cytochrome cholesterol side-chain cleavage enzyme
